# Use of FOLFIRINOX or Nab-Paclitaxel Plus Gemcitabine for the Treatment of Locally Advanced Pancreatic Adenocarcinoma: A Single Institution Observational Study

**DOI:** 10.3390/cancers13194939

**Published:** 2021-09-30

**Authors:** Alberto Servetto, Antonio Santaniello, Fabiana Napolitano, Francesca Foschini, Roberta Marciano, Eleonora Mozzillo, Priscilla Cascetta, Anna Rita Amato, Maria Rosaria Augurio, Lucia Maresca, Pietro De Placido, Sabino De Placido, Luigi Formisano, Roberto Bianco

**Affiliations:** 1 Department of Clinical Medicine and Surgery, University of Naples “Federico II”, 80131 Naples, Italy; alberto.servetto@unina.it (A.S.); santaniellanto@gmail.com (A.S.); fabiana.napolitano@unina.it (F.N.); francesca_foschini@hotmail.it (F.F.); eleonoramozzillo@libero.it (E.M.); priscillacascetta@gmail.com (P.C.); annarita.amato93@gmail.com (A.R.A.); maraaugurio@gmail.com (M.R.A.); luciamare309@gmail.com (L.M.); pietrodep91@gmail.com (P.D.P.); deplacid@unina.it (S.D.P.); 2 Department of Medicine, University of Salerno, Azienda Ospedaliera Universitaria San Giovanni di Dio Ruggi d’Aragona, 84125 Salerno, Italy; roberta.marciano@unina.it

**Keywords:** FOLFIRINOX, nab-paclitaxel plus gemcitabine, locally advanced pancreatic adenocarcinoma

## Abstract

**Simple Summary:**

We performed a retrospective analysis to evaluate the effect of treatment with FOLFIRINOX (FFN) or Nab-paclitaxel plus gemcitabine (GemNab) in patients with locally advanced (LA) pancreatic adenocarcinoma (PDAC). Forty-eight percent of patients treated with FFN became eligible for radical resection, and twenty-two percent of patients receiving GemNab underwent surgery after neoadjuvant treatment. FFN treatment was associated with a better overall survival, compared with GemNab (mOS 85.1 vs. 54.3 weeks, FFN and GemNab, respectively; HR = 0.54; *p* = 0.0109). We found different toxicity profiles between the two chemotherapy regimens. Future randomized clinical trials are mandatory to clarify the best treatment in patients with LA PDAC.

**Abstract:**

Patients with locally advanced (LA) pancreatic ductal adenocarcinoma (PDAC) do not present distant metastases but are not eligible for surgery upfront. Chemotherapy regimens, such as FOLFIRINOX (FFN) or nab-paclitaxel plus gemcitabine (GemNab) in combination with loco-regional treatments are generally used in this setting. However, the best treatment choice is unknown. We retrospectively analyzed the information of 225 patients with stage II–III PDAC treated at our institution between October 2011 and December 2020. A total of 94 patients with LA PDAC who are non-eligible for surgery upfront received neoadjuvant FFN or GemNab. Of the 67 patients receiving FFN, 28 (41.8%) underwent surgery after neoadjuvant therapy. Of the 27 patients treated with GemNab, 6 (22.2%) became eligible for resection. The median overall survival (OS) was 85.1 weeks and 54.3 weeks in the FFN and GemNab groups, respectively (HR = 0.54, *p* = 0.0109). The median OS was 189.7 weeks and 76.4 weeks in the resected and unresected cohorts, respectively (HR = 0.25, *p* < 0.0001). Neutropenia (37.3%), anemia (6.0%), and diarrhea (6.0%) in the FFN group and neutropenia (22.2%) and thrombocytopenia (18.5%) in the GemNab groups were the most frequent grade 3–4 side effects. Higher rates of thrombocytosis (*p* < 0.0001) and peripheral edema (*p* < 0.0001) were observed in the GemNab group. Our results suggest that the use of FFN is associated with more favorable clinical outcomes than GemNab for patients with LA PDAC. Future randomized and controlled clinical trials are needed to further elucidate the role of these regimens and loco-regional treatments in this setting.

## 1. Introduction

Pancreatic Ductal Adenocarcinoma (PDAC) is the fourth most prevalent cause of cancer-related death among both men and women in the USA [1]. The incidence of this cancer has surged in the last decade due to the increased spread of risk factors such as smoking, obesity, and inappropriate dietary habits [2,3]. Life expectancy for pancreatic cancer patients is generally very low, with a 5-year survival rate < 10% [4]. Despite the high incidence and severe mortality rates, the available therapeutic strategies are very limited, making the treatment of this cancer an unmet clinical need [5]. Surgical resection of pancreatic cancer represents the only valid therapeutic option able to guarantee a prolonged survival. However, only less than 20% of patients have a resectable tumor at diagnosis [6]. In patients with stage IV pancreatic cancer, the standard of care is represented by chemotherapy: the two regimens FOLFIRINOX (FFN) and nab-paclitaxel plus gemcitabine (GemNab) have shown significant improvement in progression-free survival (PFS) and overall survival (OS) compared with gemcitabine as a single-agent [7,8]. A large fraction of newly diagnosed PDACs, localized to the pancreas or pancreatic region cannot be resected upfront, mainly due to extensive vascular involvement. These stage II–III pancreatic cancers are generally divided in two categories: borderline resectable (BR) or locally advanced (LA) pancreatic cancer [9,10,11]. The best treatment strategy for LA PDACs has not been identified yet [12]. These patients undergo a chemotherapy treatment as a neoadjuvant strategy, with the aim of downsizing the primary lesion in order to select patients for radical surgery. The use of chemotherapy, radiotherapy, and other locoregional treatment strategies (i.e., radiofrequency ablation) has been investigated to treat BR and LA pancreatic cancers, with varied results [13,14,15,16]. Herein, we present a retrospective analysis of the clinical data collected from patients with LA PDAC treated at our institution. This study aimed to describe the rate of patients who underwent surgery after neoadjuvant treatment with FFN or GemNab ± locoregional therapeutic approaches. We also report an evaluation of survival outcomes of this cohort of patients as well as adverse events induced by the two chemotherapy regimens.

## 2. Methods

### 2.1. Study Design

This retrospective investigation analyzed clinical data from patients treated between October 2011 and December 2020 at our institution, the Department of Clinical Medicine and Surgery, Oncology Division, University of Naples “Federico II,” Italy. All patient information was recorded in an internal computer database. The retrospective study protocol was approved by the institutional review board at the main study site (“Federico II” University Hospital Institutional Ethics Committee, Naples; approval number: 222/21). The study was performed in accordance with the Declaration of Helsinki and Good Clinical Practice guidelines. All patients signed a written informed consent and agreed with the research use of their anonymized data. Patients received one of the following schedules: FOLFIRINOX (Oxaliplatin 85 mg/m^2^, Calcium Levofolinate 200 mg/m^2^, Irinotecan 180 mg/m^2^, 5-fluorouracile 400 mg/m^2^ bolum, and 5-fluorouracile 2400 mg/m^2^ continuous infusion 46 h d1 q14); nab-paclitaxel plus gemcitabine (Nab-paclitaxel 125 mg/m^2^ and gemcitabine 1000 mg/m^2^ d1, 8, 15 q28). Treatment choice was decided based on patients’ comorbidities and after meticulous discussion with patients about potential benefits and toxicities. Some of the patients received local radiotherapy or Radiofrequency Ablation (RFA) after neoadjuvant chemotherapy and before surgery, as discussed in the Section 3 of this paper. Data regarding adverse events were collected and graded based on the National Cancer Institute Common Terminology Criteria for Adverse Events (NCI CTCAE), version 5.0, except the evaluation of peripheral edema and thrombocytosis, for which we only evaluated presence versus absence.

### 2.2. Inclusion Criteria

In our analysis, we only considered patients with the following characteristics: pathologically confirmed diagnosis of pancreatic adenocarcinoma; age ≥ 18 years; locally advanced PDAC, based on computed tomography (CT) or magnetic resonance imaging (MRI), and criteria defining resectability status at diagnosis reported in the NCCN guidelines [17]; Eastern Cooperative Oncology Group performance status (ECOG PS) 0-1; at least 4 complete cycles of FFN or 2 cycles of GemNab; and no prior systemic or locoregional therapy for PDAC. All of the data regarding the stage of the disease were evaluated through CT scan and/or MRI. The stage of disease was defined based on the 7th edition of the American Joint Committee on Cancer (AJCC) manual [18]. All patients with radiological evidence of metastatic disease or malignant ascites were not included in the analysis.

### 2.3. Efficacy and Survival Outcomes

Response to treatment was evaluated based on Response Evaluation Criteria in Solid Tumours (RECIST) criteria v1.1. Re-assessment of the disease was performed every 4–6 cycles of FFN or 3 cycles of GemNab, and/or at the end of the neoadjuvant treatment. After surgery, routine follow-up was performed with CT scan and/or MRI, serum CA19.9 measurement, and clinical visit. Overall survival (OS) was calculated from the date of cycle 1 of neoadjuvant chemotherapy to the date of death from any cause.

### 2.4. Statistical Analysis

Survival curves were generated based on the Kaplan–Meier method. Statistical significance of survival curves was calculated using the Log-rank test. Graphpad Prism v8.0 was used to generate survival curves and to calculate statistics throughout the entire manuscript. A *p* value of less than 0.05 was considered statistically significant. A multivariate Cox regression analysis was performed to assess the relation between OS and the variables “treatment group” and “age at inclusion (years)”. The data were checked for multicollinearity with the Belsley–Kuh–Welsch technique, and proportional hazards were checked according to Schoenfeld residuals. The alpha risk was set to 5%. Statistical analysis was performed with the online application EasyMedStat (version 3.9; www.easymedstat.com, accessed date: 29 July 2021).

## 3. Results

### 3.1. Patients’ Characteristics and Treatment

**Figure 1:**
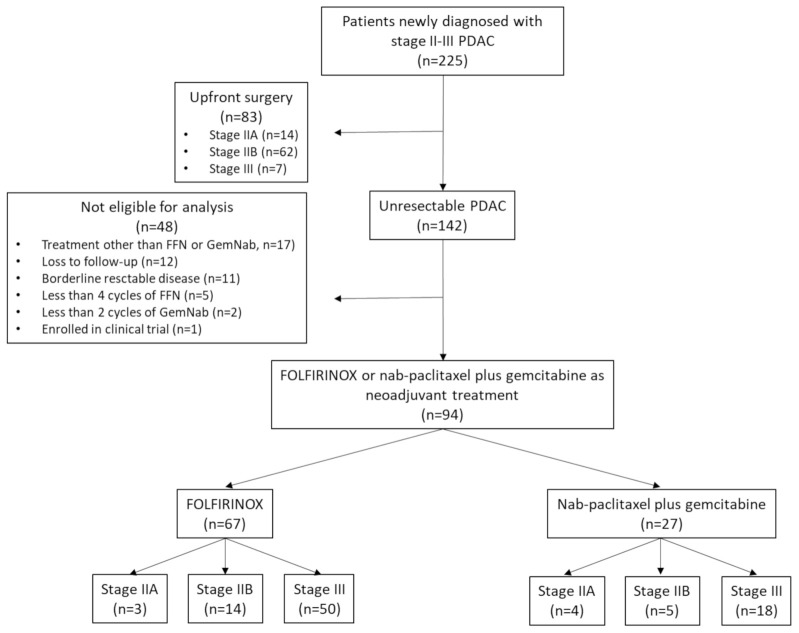
Flowchart of patient selection for retrospective evaluation.

Between October 2011 and December 2020, we evaluated 225 patients with newly diagnosed stage II–III pancreatic cancer at the Oncology Unit of the University of Naples “Federico II”, Italy. Of these, 83 patients underwent radical surgery upfront. Among the 142 unresectable cases of PDAC, we selected 67 cases treated with FFN and 27 patients receiving GemNab as neoadjuvant therapeutic approaches for further analysis (Figure 1). Only patients with locally advanced PDAC, PS ECOG 0-1, who received at least four cycles of FFN or at least two cycles of GemNab were evaluated. Thirty-six patients were not included in the analysis due to the following: treatments other than FFN or GemNab (*n* = 17); loss to follow-up *(n* = 12); administration of less than four cycles of FFN (*n* = 5) or less than two cycles of GemNab (*n* = 2); or enrollment in clinical trials (*n* = 1). In addition, 11 patients were not included because they received a diagnosis of BR PDAC. Of the 67 patients with LA PDAC treated with FFN, 3 had a diagnosis of stage IIA (*n* = 3/67, 4.5%), 14 had stage IIB (*n* = 14/67, 20.9%), and 50 had stage III (*n* = 50/67, 74.6%) PDAC. Of the 27 patients treated with GemNab, 4 had a diagnosis of stage IIA PDAC (*n* = 4/27, 14.8%), 5 had stage IIB (*n* = 5/27, 18.5%), and 18 had stage III (*n* = 18/27, 66.7%). All patients’ baseline demographic and disease characteristics are reported in Table 1. The median ages at inclusion in the study were 59.2 years and 67.8 years in the FFN and GemNab groups, respectively (*p* = 0.0003, Table 1).

### 3.2. Efficacy of Neoadjuvant Treatment

Of the 67 patients who received FFN as a neoadjuvant treatment, we registered 31 partial responses (PR, 46.3%), 21 cases of stable disease (SD, 31.3%), and 15 cases of progressive disease (PD, 22.4%) (Table 2). Instead, of the 27 patients receiving GemNab treatment, 10 experienced PR (37.1%), 6 experienced SD (22.2%), and 11 experienced PD (40.7%) (Table 2). A higher Disease Control Rate (DCR, PR + SD) was registered in the FFN group (FFN vs. GemNab, 77.6% vs. 59.3% respectively). However, no statistical difference was noted between the two treatments in terms of tumor responses (Table 2). After treatment with FFN, 28 patients (28/67, 41.8%) were considered eligible for radical surgery (Table 3). In the GemNab group, six patients (6/27, 22.2%) underwent surgery after systemic treatment (Table 3). The difference in rates of patients becoming eligible for surgery for the two groups was not statistically significant (*p* = 0.097), although a higher rate of patients undergoing surgery was found for the FFN group. In the FFN group, none of the patients with stage IIA, 9/14 (64.2%) patients with stage IIB, and 19/50 (38.0%) patient with stage III PDAC underwent surgery (Table 4). Instead, resection was performed in 2/4 (50.0%) stage IIA, 1/5 (20.0%) stage IIB, and 3/18 (18.75%) stage III PDAC patients treated with GemNab (Table 4). The patients underwent a median of nine cycles of FFN (range 4–15), and a median number of cycles of GemNab of five (range 2–16). Before surgery, the median duration of treatment was 20.0 weeks (range 6.42–35.0) in the FFN group and 20.0 weeks (range 6–66.28) in the GemNab group. In the FFN group, of the 28 patients undergoing resection, 4 patients received stereotactic body radiation therapy (SBRT), 2 radiofrequency ablation (RFA), and 1 intensity-modulated radiation therapy (IMRT) after completion of chemotherapy and before surgery (Figure S1). In the GemNab group, three out of six patients received SBRT after neoadjuvant chemotherapy but before radical surgery (Figure S1). Fifty-six patients receiving FFN (56/67, 83.6%) had delays or reduced doses of chemotherapy (range of dose reduction, 0–30%). In the GemNab group, 18 (18/27, 66.6%) patients experienced delays or dose reduction (range of dose reduction, 0–50%). Of the 39 patients receiving neoadjuvant FFN and not eligible for surgery, 19/39 (48.7%) received radiotherapy, chemo-radiation, or other locoregional treatments; 12/39 (30.8%) started a second-line chemotherapy regimen; and 8/39 (20.5%) were eligible only for best supportive care (BSC) (Supplementary Figure S1). On the other hand, among the 21 patients not suitable for resection after receiving neoadjuvant GemNab, 7/21 (33.3%) were eligible for radiotherapy, chemo-radiation, or other locoregional treatments; 11/21 (52.4%) received another chemotherapy regimen; and 3/21 (14.3%) were offered BSC. Of the 28 patients undergoing resection after neoadjuvant FFN, 15 (15/28, 53.6%) also received adjuvant chemotherapy after surgery. Of these 15 patients, 3 also received concomitant radiation. Only 1/28 resected patients received radiotherapy alone as adjuvant treatment. Of the six patients resected after neoadjuvant GemNab, three (50%) subsequently received adjuvant chemotherapy, of which two also received concomitant radiotherapy.

**Figure 2:**
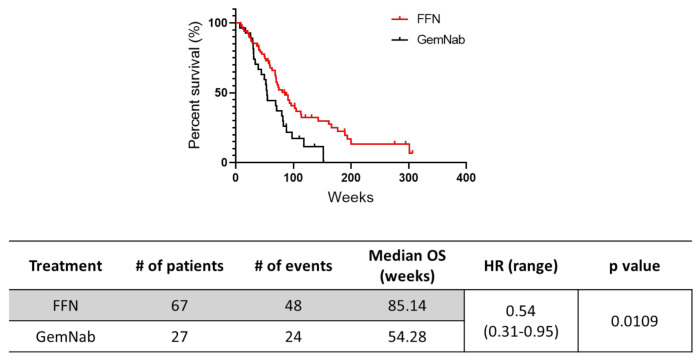
Kaplan–Meier curves for overall survival (OS) in 94 patients with locally advanced (LA) PDAC treated with either FOLFIRINOX (FFN) (*n* = 67) or nab-paclitaxel + gemcitabine (GemNab) (*n* = 27). # = number.

The median OS was 85.1 weeks in the overall population receiving neoadjuvant FFN and 54.3 weeks in the group treated with GemNab (*p* = 0.0109, HR = 0.54, 95% CI 0.31–0.95) (Figure 2). As shown in Table 1, we observed a statistically significant difference in age at diagnosis between FFN and GemNab groups. Hence, we performed a multivariate Cox regression analysis to assess the relation between the OS advantage in the FFN group and the age at inclusion in the study (Table 5). This analysis revealed that the statistically significant difference in age between the two groups (reported in Table 1) did not affect the OS data reported in Figure 2.

**Figure 3:**
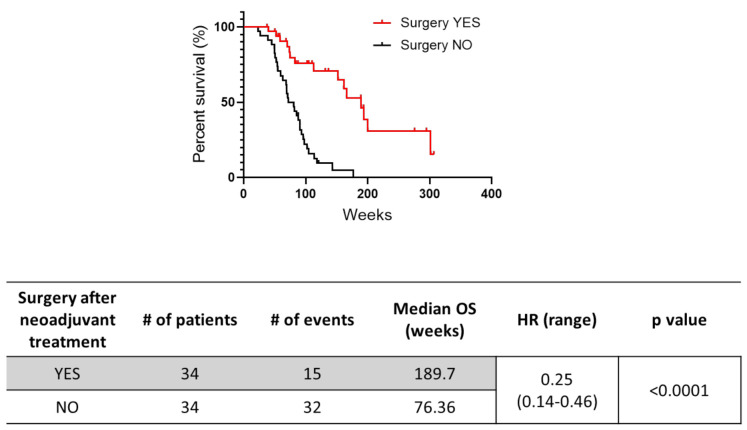
Kaplan–Meier curves for overall survival (OS) in patients who underwent surgery (*n* = 34) vs. patients who were not eligible for resection (*n* = 34) after neoadjuvant chemotherapy. Only patients who experienced partial response (PR) or stable disease (SD) after neoadjuvant treatment were considered for this analysis. # = number.

Next, we aimed to evaluate the impact of surgery (performed after neoadjuvant chemotherapy) on OS. The median OS was 189.7 weeks in the 34 patients undergoing surgery after neoadjuvant treatment (28 FFN, 6 GemNab) and 76.4 weeks in the 34 patients who did not become eligible for surgery (*p* < 0.0001, HR = 0.25, 95% CI 0.14–0.46) (Figure 3). Of note, in this latter group, we only included the patients that had a partial response or stable disease after systemic treatment and excluded patients who experienced disease progression.

### 3.3. Adverse Events

The FFN and GemNab regimens had different toxicity profiles. The most frequent grade 3-4 adverse events in the FFN groups were neutropenia (25/67, 37.3%), anemia (4/67, 6.0%), and diarrhea (4/67, 6.0%). The most common grade 3–4 toxicities in the GemNab group were neutropenia (6/27, 22.2%) and thrombocytopenia (5/27, 18.5%). We observed a statistically significant higher rate of nausea (*p* = 0.037) and peripheral neuropathy (*p* = 0.022) in the FFN group. Instead, higher rates of anemia (*p* = 0.0105), thrombocytosis (*p* < 0.0001), and peripheral edema (*p* < 0.0001) were observed in the GemNab group. All of the adverse events occurring in the two groups are listed in Table 6.

## 4. Discussion

We show herein one of the largest retrospective analyses of patients with a diagnosis of LA PDAC receiving neoadjuvant chemotherapy at our institution. FFN and GemNab are the chemotherapy schedules commonly accepted as the best systemic therapeutic approach for metastatic PDAC [7,8]. However, the recommendations about the optimal treatment strategy for patients with unresectable pancreatic cancer are generally weak. Indeed, substantial evidence, represented by randomized and controlled phase III clinical trials, is missing in this setting. A systematic review of the studies testing the efficacy of FFN in unresectable PDAC revealed an OS range between 10.0 and 32.7 months, with a patient-level pooled median OS of 24.2 months [19]. Our results are concordant with these findings, with a median OS of patients receiving neoadjuvant FFN of ~21 months (85.1 weeks). Suker and colleagues calculated that the proportion of pooled patients across 12 studies who underwent resection after FFN treatment was 25.9% [19]. The recent results of the LAPACT trial revealed that 16% of LAPC patients receiving nab-paclitaxel plus gemcitabine upfront may become eligible for surgery [20]. We acknowledge that a comparison between these different studies is methodologically incorrect, mainly due to the different selection criteria across the various studies. However, our results confirm a potentially higher conversion surgery rate in patients receiving FFN compared with GemNab (41.8% vs. 22.2%). Randomized phase III trials, with objective and standardized selection criteria, may further clarify this feature [12]. Patients with borderline resectable tumors have a higher chance of undergoing resection after neoadjuvant treatment than LA PDAC [21,22]. Hence, we excluded data from 12 patients who were classified in the group of BR PDAC from our analysis.

We acknowledge that, in our cohort, a statistically significant difference has been identified in the median age at diagnosis between FFN and GemNab. Due to a high rate of G3-4 adverse events, such as neutropenia, FFN is generally avoided in older patients, often affected by other comorbidities [23]. However, our results indicate that the different ages of the patients in the two groups at the time of the start of neoadjuvant treatment did not affect the OS results reported in Figure 2, in which it is shown that the FFN treatment is associated with better survival outcomes. Our results also suggest that the better OS registered in the FFN group (Figure 2) may be due to the higher rate of radical surgery in the patients treated with this regimen (41.8% vs. 22.2%, Table 3). Of note, stages II–III were well balanced between the two groups, with a higher prevalence of stage III at diagnosis. However, the stage of disease did not predict the chance to be selected for surgery after neoadjuvant treatment (Table 4). This is consistent with the fact that resectability criteria are strongly dependent on vascular involvement, more than stage of disease.

A large fraction of the patients involved in this study received radiotherapy, chemo-radiation, or other locoregional approaches. Although chemoradiation is routinely performed for unresectable PDAC, its efficacy is still controversial. SBRT is generally accepted as a safe strategy to increase the rate of resections in patients with borderline resectable or locally advanced PDAC [24]. Chen and colleagues revealed that combined treatment strategies might guarantee better results compared with radiotherapy alone [25]. However, chemo-radiation was not associated with improvements in survival outcomes and was burdened by an increased rate in grade 3–4 hematological and non-hematological toxicities [25].

The role of additional systemic treatment after surgery in patients who had already received neoadjuvant therapy is not clear [17]. Of the 34 patients evaluated in our study who underwent resection, 18 (52.9%) received postoperative chemotherapy. Further controlled and more extensive studies are needed to better elucidate the role of chemotherapy in this setting and its impact on rates of relapse and survival.

We acknowledge that our study has several limitations. Indeed, this is a retrospective and non-randomized study, with intrinsic bias. The treatments were decided by physicians, after discussion with patients and not through a randomization system. Next, the use of locoregional treatments may have affected the results, both on resectability and survival outcomes. These features, in addition to the small sample size of the cohorts, do not allow for drawing definitive conclusions about the best therapeutic strategy for LA PDAC.

In our cohort, the toxicity profiles of FFN and GemNab regimens were not inconsistent with previous reports [26,27]. In both groups, neutropenia was the most frequent grade 3–4 adverse event. All of these patients were treated with granulocyte colony-stimulating factor (G-CSF) [28]. Interestingly, we found a significant enrichment of thrombocytosis and peripheral edema in patients treated with GemNab. Few data have been previously published regarding a possible association of nab-paclitaxel plus gemcitabine treatment and thrombocytosis [29]. These findings may suggest further investigation of the potential risks of thromboembolic events in pancreatic cancer patients treated with GemNab. Indeed, a careful pretreatment assessment of venous thromboembolism risk [30] may also need to consider the potential risk of thrombocytosis induced by GemNab.

## 5. Conclusions

Our retrospective study shows that FFN should be considered the optimal chemotherapy regimen to be used in patients with locally advanced PDAC. Future randomized studies may clarify whether one of the two schedules is associated with better survival outcomes. We recommend that a multidisciplinary assessment should always be performed to establish the best treatment strategy for these patients.

## Figures and Tables

**Table 1:** Baselines of patient characteristics.

Characteristics of Patients	FOLFIRINOX (*n* = 67)	Nab-Paclitaxel + Gemcitabine (*n* = 27)	*p*
Age at inclusion, Median (range)	59.2 (35.4–74.9)	67.8 (43.8–77.4)	0.0003
Sex Male Female	34 33	14 13	>0.99
Site (*n*): Head Body/Tail	42 25	21 6	0.23
PS ECOG: 0 1	58 9	23 4	>0.99
Basal CA19.9, UI/mL ≤37 >37 N/A	17 47 3	3 21 3	0.19
Staging IIA IIB III	3 14 50	4 5 18	0.22
T classification: T2 T3 T4	4 13 50	0 9 18	0.18
N classification: N0 N+ N/A	14 45 8	7 15 5	0.54

**Table 2:** Outcome after neoadjuvant treatment in patients receiving either FOLFIRINOX or nab-paclitaxel + gemcitabine as a neoadjuvant treatment.

Outcome after Neoadjuvant Treatment	FOLFIRINOX (%) (*n* = 67)	Nab-Paclitaxel + Gemcitabine (%) (*n* = 27)	*p*
Partial Response (PR)	31 (46.3)	10 (37.1)	0.19
Stable Disease (SD)	21 (34.3)	6 (22.2)	
Progressive Disease (PD)	15 (22.4)	11 (40.7)	

**Table 3:** Rates of patients who became eligible for surgery after neoadjuvant treatment.

Surgery	FOLFIRINOX (%) (*n* = 67)	Nab-Paclitaxel + Gemcitabine (%) (*n* = 27)	*p*
YES	28 (41.8)	6 (22.2)	0.097
NO	39 (58.2)	21 (77.8)	

**Table 4:** Classification of patients undergoing surgery after neoadjuvant FOLFIRINOX or nab-paclitaxel + gemcitabine, based on the stage of PDAC.

Stage	FOLFIRINOX	Nab-Paclitaxel + Gemcitabine
IIA Resected Total	0 3	2 4
IIB Resected Total	9 14	1 5
III Resected Total	19 50	3 18

**Table 5:** A multivariate Cox regression analysis to assess the relation between OS and the variables “treatment group” and “age at inclusion (years)”. The hazard ratio (HR) for each increase of 1 unit of age at inclusion (years) was 1.02 (95% CI: [0.987; 1.05], *p* = 0.275). Compared with patients in the FFN group, the HR was 1.78 ([1.03; 3.07], *p* = 0.04) for patients in the GemNab group. Statistical analysis was performed as described in the Section 2.

Variables	Hazard Ratio	*p*
Treatment Group Reference: FFN GemNab		
	1.78 [1.03; 3.07]	0.04
Age at Inclusion (years) Risk for each 1-unit increase		
	1.02 [0.987; 1.05]	0.274

**Table 6:** Adverse events associated with either neoadjuvant FOLFIRINOX or nab-paclitaxel + gemcitabine.

Adverse Event	FOLFIRINOX *n* = 67 (%)		Nab-Paclitaxel + Gemcitabine *N* = 27 (%)	
	G1/2	G3/4	G1/2	G3/4
Anemia	45 (67.2)	4 (6.0)	23 (85.2)	3 (11.1)
Neutropenia	23 (34.3)	25 (37.3)	12 (44.4)	6 (22.2)
Thrombocytopenia	41 (61.2)	2 (1.5)	13 (48.1)	5 (18.5)
Increased AST and/or ALT levels	34 (50.8)	3 (4.5)	16 (59.3)	3 (11.1)
Oral Mucositis	13 (19.4)	0 (0)	5 (18.5)	0 (0)
Diarrhea	33 (49.3)	4 (6.0)	12 (44.4)	2 (7.4)
Nausea	43 (64.2)	1 (1.5)	11 (40.8)	0 (0)
Vomiting	24 (35.8)	1 (1.5)	5 (18.5)	0 (0)
Fatigue	51 (76.1)	0 (0)	20 (74.1)	1 (3.7)
Periferal neuropathy	40 (59.7)	1 (1.5)	6 (22.2)	3 (11.1)
Thrombocytosis (platelet count > 500 × 10^3^)	1 (1.5)		11 (40.7)	
Peripheral edema	1 (1.5)		8 (29.6)	

## Data Availability

The data presented in this study are available from the corresponding author upon reasonable request.

## Supplementary Materials

The following are available online at https://www.mdpi.com/article/10.3390/cancers13194939/s1, Figure S1. Flowchart of patients evaluated in this study based on surgery eligibility and locoregional treatments.
