# Supplementary figures and images for: Use of FOLFIRINOX or Nab-Paclitaxel Plus Gemcitabine for the Treatment of Locally Advanced Pancreatic Adenocarcinoma: A Single Institution Observational Study
Source: Cancers (Basel). 2021 Sep 30;13(19):4939. doi: 10.3390/cancers13194939 (PMC8508515; doi:10.3390/cancers13194939)

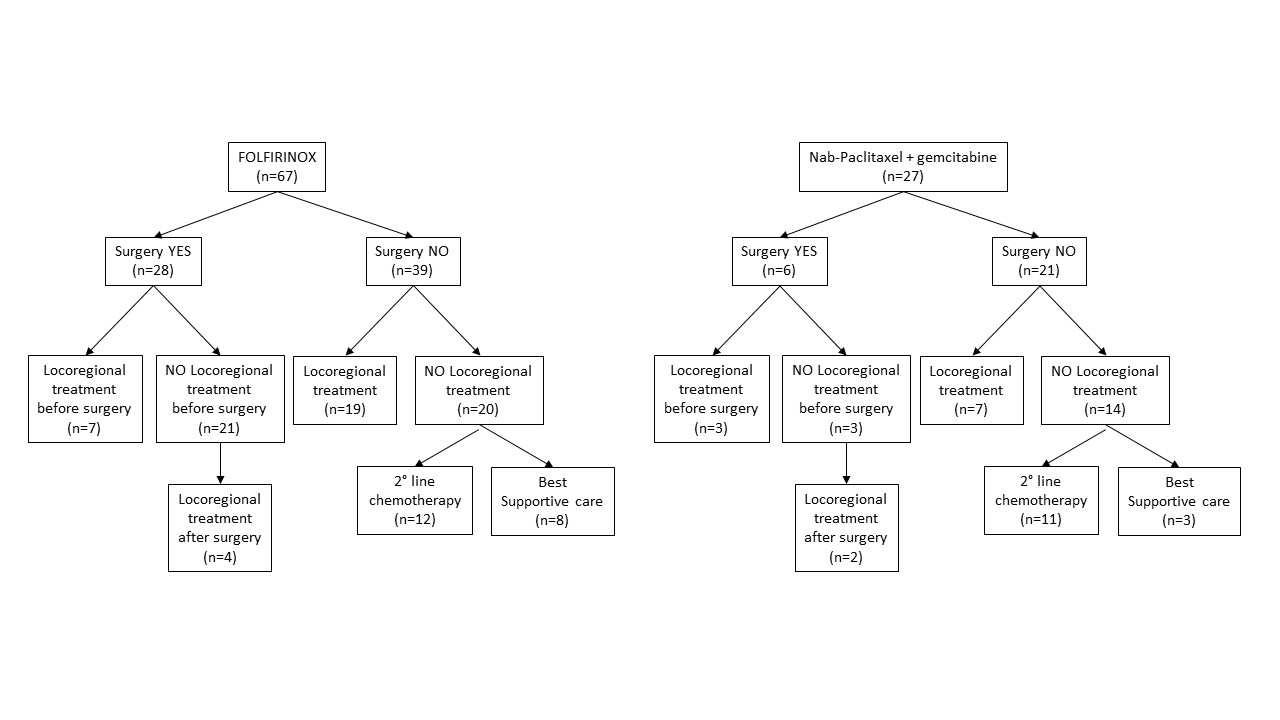

Supplement: Supplementary file 1 [file cancers-13-04939-s001.zip › cancers-1380891-supplementary.jpg]
